# The Influence of Internal Packaging (Liners) on Moisture Dynamics and Physical and Physiological Quality of Pomegranate Fruit during Cold Storage

**DOI:** 10.3390/foods10061388

**Published:** 2021-06-16

**Authors:** Robert Lufu, Alemayehu Ambaw, Umezuruike Linus Opara

**Affiliations:** 1 SARChI Postharvest Technology Research Laboratory, Africa Institute for Postharvest Technology, Faculty of AgriSciences, Stellenbosch University, Stellenbosch 7602, South Africa; lufurobert@gmail.com (R.L.); tsige@sun.ac.za (A.A.); 2 UNESCO International Centre for Biotechnology, Nsukka 410001, Nigeria

**Keywords:** internal packaging, modified atmosphere packaging, storage quality, transpiration, water loss

## Abstract

Weight loss and decay are common physiological disorders during postharvest handling and storage of pomegranates. The study focused on relating the ability of plastic liners as internal packaging to modify both gaseous and moisture atmosphere around the fruit to moisture dynamics and physical and physiological quality of pomegranate fruit (cv. Wonderful) during storage. Fruit were packed with no-liner, non-perforated ‘Decco’, non-perforated ‘Zoe’, micro-perforated Xtend^®^, 2 mm macro-perforated high density polyethylene (HDPE), and 4 mm macro-perforated HDPE plastic liners. After 84 days of storage at 5 °C and 90–95% relative humidity (RH), fruit packed with no-liner lost 15.6 ± 0.3% of initial weight. Non-perforated (Decco and Zoe) liners minimised losses to 0.79 and 0.82% compared to Xtend^®^ micro-perforated (4.17%) and 2 mm HDPE (2.44%) and 4 mm macro-perforated HDPE (4.17%) liners, respectively. Clearly, micro- and macro-perforation of liners minimised moisture condensation, fruit decay, and shrivel severity. Micro-perforated Xtend^®^ and macro-perforated 4 mm HDPE were the best treatments in minimising postharvest losses that are often associated with inadequate environment control inside packaging compared to the use of non-perforated liners.

## 1. Introduction

Production and consumption of pomegranate (*Punica granatum* L.) fruit is on the increase worldwide. The fruit has an edible portion of about 55–60% [1] and can be eaten fresh or processed into juice, wine, and jam [2,3,4]. Freshly harvested fruit is kept under cold storage awaiting export to distant markets. Fruit from South Africa takes about 42 d to reach Europe as the major export market destination and therefore a need to maintain good postharvest quality during prolonged storage and export conditions. Storing pomegranate (cv. Wonderful) for 3 months at 5 °C and above 92% relative humidity (RH) minimises physiological disorders and maintains internal and external quality attributes [5]. Chilling injury increases with storage duration and temperatures lower than 5 °C [6].

In postharvest fruit handling, weight loss and fruit decay are common physiological disorders, among others such as chilling injury and scalding, contributing to quantitative and qualitative loss [6,7]. Pomegranates are highly prone to moisture loss owing to the relatively high water permeability across the skin through minute openings, despite having a thick rind [6,8,9]. Fruit moisture loss if not well controlled results into shrinkage; shrivel; and quantitative loss in weight, taste, and overall acceptability of the fruit, and hence market loss [10].

Internal packaging techniques have been used in the fresh fruit industry to minimise moisture loss. Internal packaging refers to additional packaging materials applied around the fruit within the external package. Surface coating and waxing has been applied on apples, oranges, plums, and pomegranate to minimise moisture loss [8,11,12,13,14,15]. For pomegranates, heat shrinkable wraps on individual fruit in cartons have also been applied [8,16,17]. On the other hand, shrink wrapping, surface coating, or waxing can lead to anaerobic respiration by creating an oxygen deficit and yet promoting a high CO_2_ atmosphere around the fruit. This results in the production of off flavors and a change in taste [18,19]. Plastic liners are a commonly applied internal packaging to minimise moisture loss for pomegranate fruit packaged in ventilated cartons [20,21,22]. Previous research has reported their ability to modify gaseous atmosphere around the fruit, preserving physical and physio-chemical quality [16,22]. However, non-perforated liners negatively affect the fruit cooling rate and increase energy usage during forced air-cooling operations [23,24]. The authors investigated the effect of individual carton design, stack orientation, and presence of internal packaging liners. The internal liner was identified as the most significant factor influencing the produce cooling rate, increasing the seven-eighth cooling time by more than twofold and the corresponding energy usages by up to threefold compared to stacks with no liners. In addition, non-perforated liners promote moisture condensation and consequential fruit decay. Mphahlele et al. [20] reported a higher decay incidence of 33.9% in fruit packed inside plastic liners compared to 29.2 and 16.7% for fruit packed in shrink-wraps and open cartons, respectively, by the end of three months of storage at 7 °C and 90% RH.

The use of perforations is identified as a solution to minimise moisture condensation on fruit surfaces and liner walls, thus minimising the consequential fruit decay as a result of the improved vapor transmission capabilities of the perforated liners [25,26]. However, there is still very limited literature on the effect of liner on the keeping quality of pomegranates. Specifically, perforated liners have not been properly studied and yet have the potential to reduce quality losses during prolonged fruit storage. Therefore, the role of liner perforation in counterbalancing between minimising excessive moisture loss and fruit decay will be demonstrated during prolonged storage. The present study focused on relating the ability of plastic liners (non-perforated, micro-perforated, and macro-perforated) as internal packaging to modify both gaseous and moisture atmosphere around the fruit, to moisture dynamics and physical and physiological quality of pomegranate fruit (cv. Wonderful) during storage.

## 2. Materials and Methods

### 2.1. Fruit Supply

Commercially mature, harvested pomegranate fruit (cv. Wonderful) of uniform diameter 81.8 ± 2.5 mm and mass 286 ± 15 g were procured from a farm in Bonnievale (33°58′12.02″ S, 20°09′21.03″ E), Western Cape, South Africa. Fruit were transported in refrigerated truck to Postharvest Technology Research Laboratory at Stellenbosch University.

### 2.2. Packaging and Storage

Fruit were portioned into six treatments: no-liner (control); non-perforated ‘Decco’ liner; non-perforated ‘Zoe’ liner (ZOEpac, South Africa); micro-perforated Xtend^®^ liner; macro-perforated high density polyethylene (HDPE) liner with 54 holes of 2 mm diameter each (2 mm HDPE); macro-perforated HDPE liner with 36 holes of 4 mm diameter each (4 mm HDPE). For each treatment, 11 ventilated cartons each, loaded with 12 fruit were stored in cold rooms at 5 °C and 90–95% RH for 84 d. For each treatment, 12 fruit were randomly selected from the stack and assessed for quality after 28, 42, 56, and 84 d of cold storage.

### 2.3. Gas Analysis

A gas analyser (Checkmate 3, PBI Dansensor, Ringstead, Denmark) with a precision of ±0.5% was used to assess the gas atmosphere inside liners, across a rubber septum on the packaging film. The experiment was carried out in triplicate.

### 2.4. Water Vapor Transmission Rate (WVTR)

During storage of packaged fruit, moisture moves across films by diffusion force because of a concentration gradient created on opposite sides of the film. A modification of the dry cup technique (ASTM, 2005 method E96-95) was used to determine WVTR gravimetrically, as described by Opara et al. [27]. Film samples were cut out from areas of no-perforation on the liners in order to test for vapor transmission across the liner surface. Transmission across perforations was also assessed, and in this case film samples having one perforation at the centre were cut out from the macro-perforated liners (2 mm HDPE and 4 mm HDPE). The experiment was carried out in triplicate. Aluminium test cups were filled with 8.0 ± 0.5 g of anhydrous CaCl_2_ salt. The cups were fitted with an O-ring and grease to provide proofing against moisture and air. A film sample was then laid on top and the cup tightly closed giving an active surface area of 25 cm^2^. Cups were weighed and stored in sets under different conditions: 20 °C, 65% RH and 5 °C, 90% RH. The WVTR (g m^−2^ d^−1^) of films were calculated on the basis of mass of water gained by CaCl_2_ salt in the cup over time.

### 2.5. Condensation Assessment

Studies on moisture condensation with the five different liners were carried out in two set ups. The first set up was to determine how much visible condensate could be quantified inside the liner bags. Fruit were conditioned at ambient conditions of 17 ± 2 °C and 65 ± 5% RH for 12 h, weighed individually, packed and sealed in dozens in plastic liners, and placed inside ventilated cartons. Fruit were then stored on pallets in a cold room at 5 °C and 90 ± 5% RH for 24 h. Relative humidity and temperature of the room and inside individual carton liners was monitored using Tinytang sensors (Tinytag TV-4500, Hastings Data Loggers, Port Macquarie, Australia) at intervals of 600 s. Dry clean paper pads of known mass were used to sponge off the condensate water from the inside of the bag and on the fruit. The weight of wet pads was then immediately recorded. The amount of condensate was expressed in grams per day and as a percentage of the fruit mass. The experiment was repeated three times. The amount of condensate was also scored on a scale of 0–10 (where 0 = none; 1–2 = trace; 3–4 = slight; 5–6 = moderate; 7–8 = severe; 9–10 = extremely severe).

The second set up of the experiment was to determine the rate of change in the condensate within the bags over a period. In this case, fruit were conditioned at ambient temperatures while as the packaging material was conditioned at 5 °C in cold room for 12 h. The packed fruit were then weighed before storage at 5 °C and 90 ± 5% RH for 7 d. The condensate within the bags was scored on a 0 to 5 scale and the change in weight of the packed fruit were monitored per day. At the end of 7 d, the amount of remaining condensate in the liners was quantified as described in phase one above and the weight of fruit were also recorded. The rate of change in condensate was calculated in grams per day.

### 2.6. Weight and Size Loss Assessment

Twelve fruit were randomly selected, numbered, and monitored. The same individual fruit were monitored for weight, length, diameter, and circumference after 28, 42, 56, and 84 d of storage. Fruit weight was monitored using a digital scientific scale (Mettler Toledo, model ML3002E, Switzerland, 0.0001 g accuracy). Fruit circumference (C) was measured twice per sample fruit in the horizontal plane, using a fruit size (circumference) measurer strap band (GÜSS-FTA, Strand, South Africa). Fruit length (L) and diameter (D) were measured at two opposite longitudinal (excluding the fruit calyx) and equatorial fruit perimeters, respectively, using a digital Vernier calliper (Mitutoyo, Kawasaki, Japan, ±0.01 mm).

### 2.7. Shriveling and Decay

Incidence and severity of fruit physiological disorders of decay and shrivelling were assessed per treatment after 28, 42, 56, and 84 d of storage. The severity of each disorder was assessed subjectively using a hedonic scale of 0–5, where 0 = none; 1 = trace; 2 = slight; 3 = moderate; 4 = severe; 5 = extremely severe. Only severe injuries could be considered as commercially unacceptable [28]. Shrivel and decay indices were calculated by multiplying the scores of severity by the number of fruit affected, divided by the total number of fruit [28,29].

### 2.8. Respiration Rate

A closed system method [7] was applied to measure fruit respiration using five replicates per treatment. For each replicate, two fruit of known mass were placed inside a 3 L glass jar that was air-tight sealed with a lid with a rubber septum. The jars were incubated for 4 h at 5 °C and 90% RH. The accumulation of CO_2_ inside each glass jar was monitored using an O_2_/CO_2_ gas analyser (Checkmate 3, PBI Dansensor, Denmark) and respiration rate presented as mean ± S.E. (mL CO_2_ kg^−1^ h^−1^).

### 2.9. Fruit Puncture Resistance

The ability of the fruit to resist a penetrating force was determined by a fruit puncture analyser (GÜSS-FTA, Strand, South Africa) with a 5 mm diameter probe, as described by Arendse et al. [5]. The probe was set to penetrate 8.9 mm into the fruit at 10 mm s^−1^. The test was carried out on opposite sides of each of the 12 fruit per treatment, and the peak force (N) required to puncture the fruit was reported as puncture resistance mean ± standard error.

### 2.10. Aril Texture Analysis

Aril compression test was performed as described by Fawole and Opara [29]. Four arils were randomly chosen from each fruit segment to make a pool and then two arils selected from the pool, giving a total of 24 arils per treatment. A 35 mm diameter probe of the texture profile analyser TA. XT (Stable Micro System, Godalming, UK) was used to compress the aril at a test speed of 0.5 ms^−1^ and 0.20 N trigger force. Aril firmness was calculated as maximum force (N) required to completely break the aril. The means (±S.E.) of 24 determinations were reported per treatment.

### 2.11. Color Properties

A digital colorimeter (Minolta, model CR-400, Tokyo, Japan) was used. Fruit peel colour was monitored at two selected and ring-marked positions per fruit. Aril colour was monitored in a Petri dish at two random spots per sample. Values of L*(lightness), a* (redness), b*(yellowness), and C* (chroma) were measured. Where, a* describes surface color in the range from green (−a*) to red (+a*), while b* ranges from yellow (+b*) to blue (−b*). Monochromaticity L* ranges from 0 (black) to 100 (white). C* was calculated by equation 1 [30].Twelve replicates were considered per packaging treatment.

(1) C*=(a*2+b*2)12

### 2.12. Statistical Analysis

Analysis of variance (ANOVA) was carried out using Statistica software (Statistica 13.0, StatSoft Inc., Tulsa, OK, USA). A two-way ANOVA was applied where applicable with packaging treatments and storage time being the major categories. Means were separated using Duncan’s multiple range test, and significant difference between means was considered at *p* < 0.05. Results were presented as mean (±S.E.) of the studied variables. Relationship among selected parameters was determined by subjecting data to principal component analysis (PCA) using XLSTAT software version 2012.04.1 (Addinsoft, Paris, France).

## 3. Results and Discussion

### 3.1. Liner Properties

#### 3.1.1. Gas Composition Inside Liners

**Figure 1:**
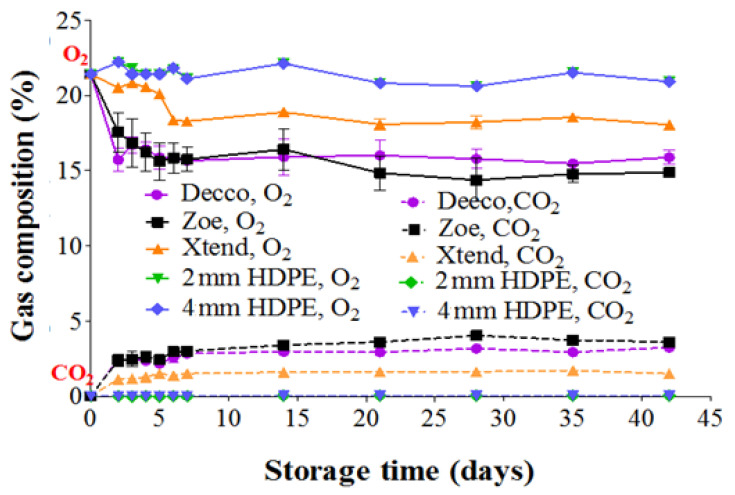
Gas composition inside plastic liners packed with pomegranate fruit (cv. Wonderful) stored at 5 °C and 90% relative humidity (RH). HDPE: high density polyethylene.

There was a decrease in O_2_ and an increase in CO_2_ composition within non-perforated ‘Decco’ and ‘Zoe’ liners and to a slight extent inside micro-perforated Xtend^®^ liners (Figure 1). Non-perorated liners provide the barrier that restricts movement of gases across packaging walls. However, there was no change in gas composition of the atmosphere inside the 2 mm macro-perforated and 4 mm macro-perforated HDPE liners. For fruit packed in non-perforated ‘Decco’ and ‘Zoe’ liners, O_2_ composition inside the liners decreased from 21.4 to 15.9 and 15.6%, respectively, while CO_2_ composition increased from 0.0 to 2.2 and 2.4%, respectively, after 5 d of cold storage. At 28 d of cold storage, CO_2_ composition further increased to 3.1% and 4.0%, inside non-perforated ‘Decco’ and ‘Zoe’ liners, respectively. After 28 d, gas composition inside non-perforated liners remained more stable. Mphahlele et al. [20] observed a more stable O_2_ concentration inside polyliners after a month of storing pomegranate (cv. Wonderful) at 7 °C. However, a steadier decrease in O_2_ and increase in CO_2_ concentrations inside different modified atmosphere packaging (MAP) liners was observed for other pomegranate cultivars (‘Hicaznar’ and ‘Hicrannar’) stored at 6 °C [21,22]. Quite similar to the current findings, Selcuk and Erkan [22] reported an increase in CO_2_ from 0.0 to 3.9 and 2.5% for pomegranate packed in MAP1 and MAP2 liners, respectively, after 20 d of storage at 6 °C.

#### 3.1.2. Water Vapor Transmission Rate (WVTR)

**Figure 2:**
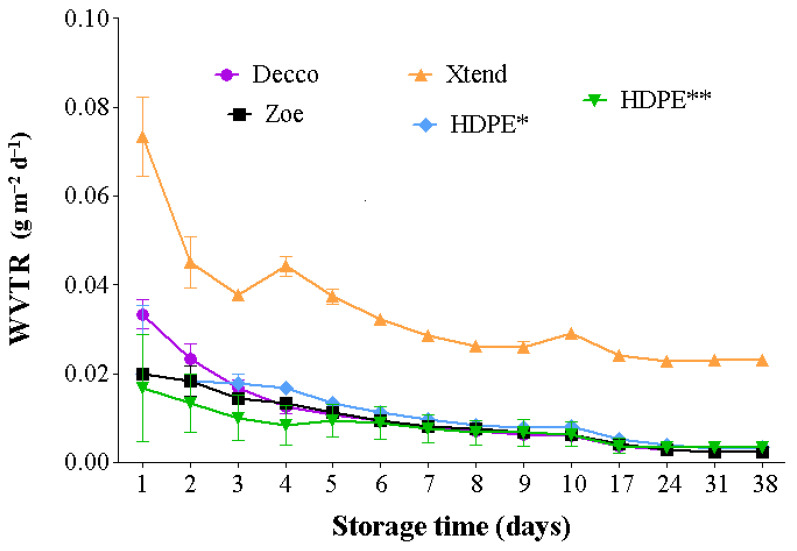
Water vapor transmission rate (WVTR) across plastic liner films under a controlled environment of 5 °C and 90% relative humidity (RH). The non-perforated film section of the 2 mm HDPE (*) and 4 mm HDPE (**) liners were used. HDPE: high density polyethylene.

**Figure 3:**
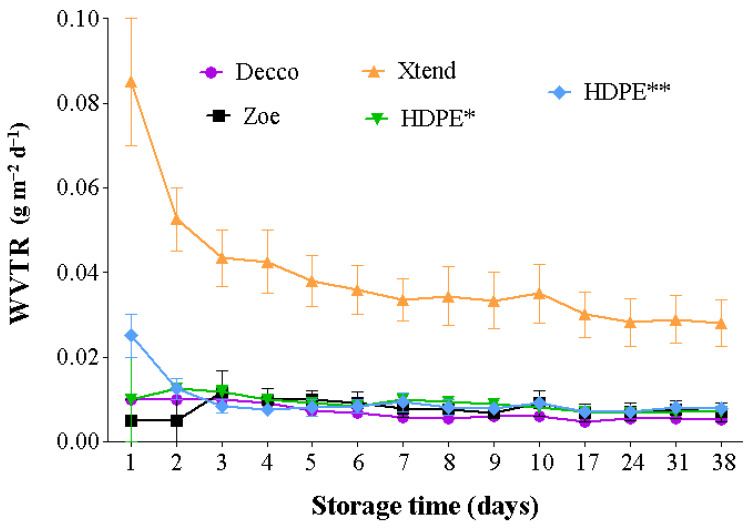
Water vapor transmission rate across plastic liner walls under a controlled environment of 20 °C and 65% relative humidity (RH). The non-perforated film section of the 2 mm HDPE (*) and 4 mm HDPE (**) liners were used. HDPE: high density polyethylene.

The rate at which a plastic liner is able to allow moisture across its walls is important in controlling humidity within the bag and around the fruit, and hence reducing condensation and associated risks of fruit decay during prolonged storage [31,32]. Water vapor transmission rate is dependent on liner permeability and prevailing storage temperatures and humidity differences inside and outside the plastic bags [27,33,34,35]. Generally, for all treatments WVTR decreased with time and then became more stable after about 15 d. Water vapor transmission rate was higher at 20 °C and 65 ± 5% RH than at 5 °C and 95% RH (Figure 2 and Figure 3). The micro-perforated Xtend^®^ liner exceptionally had a higher WVTR of 72.2 7 and 78.7 g m^−2^ d^−1^ at 5 °C and 20 °C, respectively. There was no difference in WVTR across all non-perforated films, irrespective of the type of plastic material and temperature of storage.

**Figure 4:**
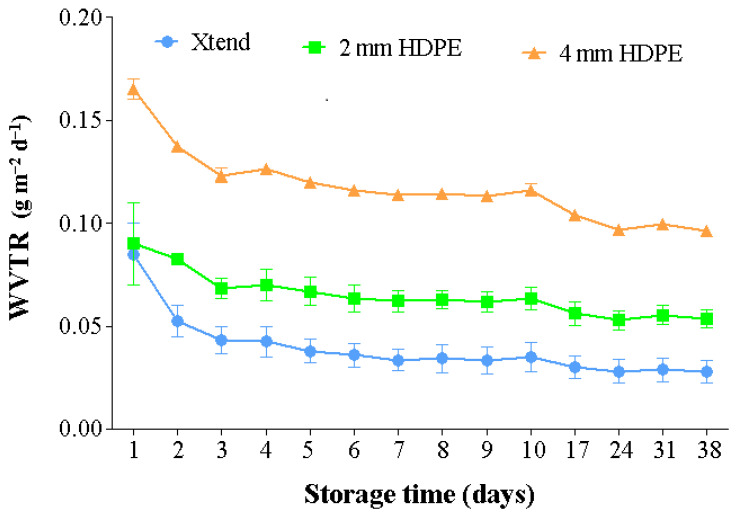
Effect of perforation on water vapor transmission rate (WVTR) under a controlled environment of 20 °C and 90% relative humidity (RH). HDPE: high density polyethylene.

Perforations improved the WVTR of the HDPE films. The presence of one 4 mm diameter perforation improved ventilation area of the HDPE film by 2.56% compared to 0.64% by one 2 mm diameter perforation. At 20 °C, the HDPE film with one 4 mm diameter perforation had 66.6% and 44.6% faster WVTR compared to micro-perforated Xtend^®^ film and HDPE film with one 2 mm diameter perforation, respectively (Figure 4). Therefore, the size of perforation plays a significant role in moisture transmission and controlling condensation within bags. Dirim et al. [33] reported a good relationship between film perforation area and WVTR at different temperature and RH conditions. Similar to our results, Opara et al. [27] observed increased WVTR with increased temperature, across biodegradable and synthetic polyfilms. The authors reported that increasing the number of perforations increased WVTR more than increasing storage temperature. Studies on water permeability across polypropyrene films showed increasing WVTR with increasing perforation diameter [34].

#### 3.1.3. Moisture Condensation Dynamics

##### One-Day Condensation Characteristics

The barrier effect of the liners permits them to retain a high RH around the fruit [36], resulting in moisture condensation. Generally, the rate of one-day condensate build-up was higher in non-perforated liner treatments than in perforated liner treatments (Table 1). Perforations improve vapor transmission capability of the liners, minimising vapor condensation inside MAP liners [25]. One-day condensate build-up was high in 2 mm macro-perforated HDPE liners, probably because of low perforation area (0.022%). However, one-day condensate build-up was lowest in micro-perforated Xtend^®^ liners and in 4 mm macro-perforated HDPE liners because of their high moisture permeability. Similarly, a higher one-day condensation severity score was observed in non-perforated liners than in perforated liners. One-day condensation severity was such that non-perforated ‘Decco’ > non-perforated ‘Zoe’ > 2 mm macro-perforated HDPE > 4 mm macro-perforated HDPE > micro-perforated Xtend^®^ liners (Table 2). A difference in the general characteristics (size and distribution) of condensate droplets formed within the different liner bags was observed (Table 2).

##### Condensation Behaviour over Prolonged Period

**Figure 5:**
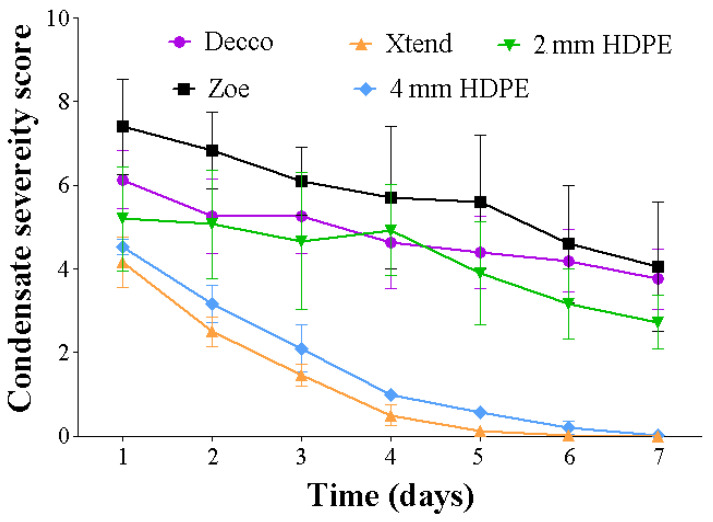
Variation of condensate inside liner bags, as depicted by 0–10 score scale (where 0 = none; 1–2 = trace; 3–4 = slight; 5–6 = moderate; 7–8 = severe; 9–10 = extremely severe). Pomegranate (cv. Wonderful) stored at 5 °C and 90% relative humidity (RH). HDPE: high density polyethylene.

**Figure 6:**
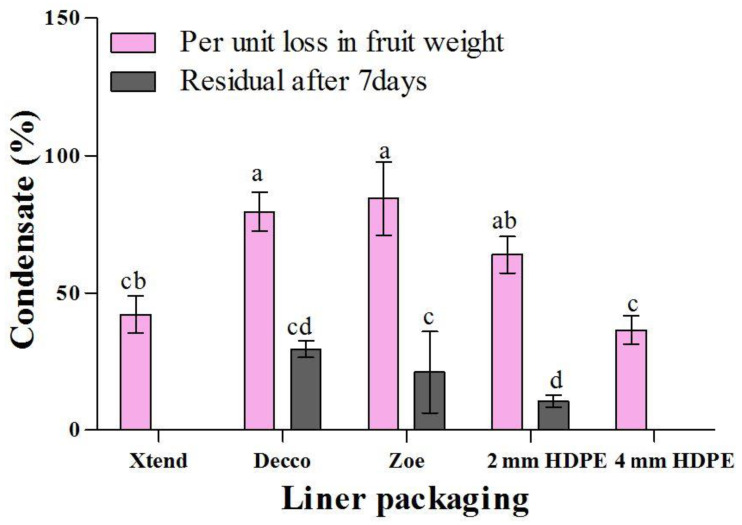
Condensate within liner bags with respect to weight lost for pomegranate (cv. Wonderful) stored at 5 °C and 90% RH for 1 d and condensate retained within plastic bags after a period of 7 d at 5 °C and 90% relative humidity (RH). Histograms columns with different letters are significantly different at *p* < 0.05 according to Duncan’s multiple range test. Vertical bars represent S.E. HDPE: high density polyethylene.

Condensate behaviour over time provides insight about water vapor transmission properties of the liners. A lower rate of condensation in the perforated liners suggests a faster moisture transmission rate across the walls of the liners, hence delayed build-up of humidity within the bags compared to non-perforated liner treatments. Severity of condensate within the bags decreased with time (Figure 5). The decrease in observable condensate was slowest in non-perforated liners compared to perforated liners. The rate at which condensate was decreasing was lowest in non-perforated ‘Zoe’ liners, followed by non-perforated ‘Decco’ liners. Condensate reduction rate was highest in micro-perforated Xtend^®^ liners, followed by 4 mm macro-perforated HDPE and 2 mm macro-perforated HDPE liners. This can be attributed to a higher water vapor transmission rate across the micro-perforated liner compared to the rest of the liners. After 3 d of monitoring, condensation severity was in traces for micro-perforated Xtend^®^ and 4 mm macro-perforated HDPE liners. By the end of 7 d of condensate monitoring, the micro-perforated Xtend^®^ and macro-perforated 4 mm HDPE liners retained none of the condensate, while macro-perforated 2 mm HDPE and non-perforated ‘Zoe’ and ‘Decco’ liners retained 10.2, 33.7, and 29.8%, respectively (Figure 6). In another study, a particular MAP liner (Xtend^®^) was reported to eradicate vapor condensation in pomegranate fruit because of its high water vapor transmission compared to polypropylene bags, which showed progressive moisture accumulation [37].

##### Condensation and Fruit Mass Loss

**Figure 7:**
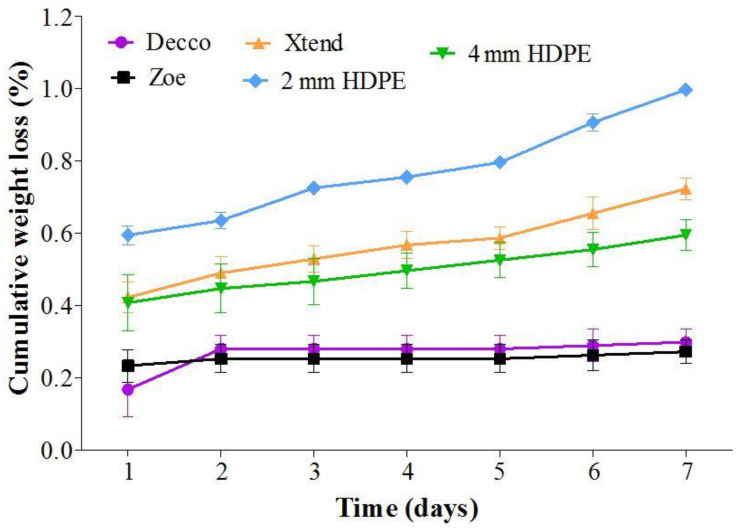
Cumulative percentage loss in weight during condensation variation within liner bags. Pomegranate (cv. Wonderful) stored at 5 °C and 90% relative humidity (RH). HDPE: high density polyethylene.

The liner treatments with a lower rate of condensate reduction (high condensate retention) had a lower rate of fruit weight loss while treatments with a higher condensate reduction had a higher rate of fruit weight loss. Fruit in non-perforated liner treatments had a lower rate of weight loss than fruit in perforated liners during the 7 d of condensate monitoring (Figure 7). Fruit weight loss is commonly a result of moisture loss, while condensation results from the moisture lost by the fruit. In non-perforated ‘Decco’ and ‘Zoe’ liners, 79.6 and 84.4% of fruit moisture loss per day was retained as condensate compared to 42.1, 63.9, and 36.4% for micro-perforated Xtend^®^, 2 mm macro-perforated HDPE liners, and 4 mm macro-perforated HDPE liners, respectively (Figure 6).

### 3.2. Weight Loss, Lineal Size Loss, and Shrivel

#### 3.2.1. Fruit Weight Loss

**Figure 8:**
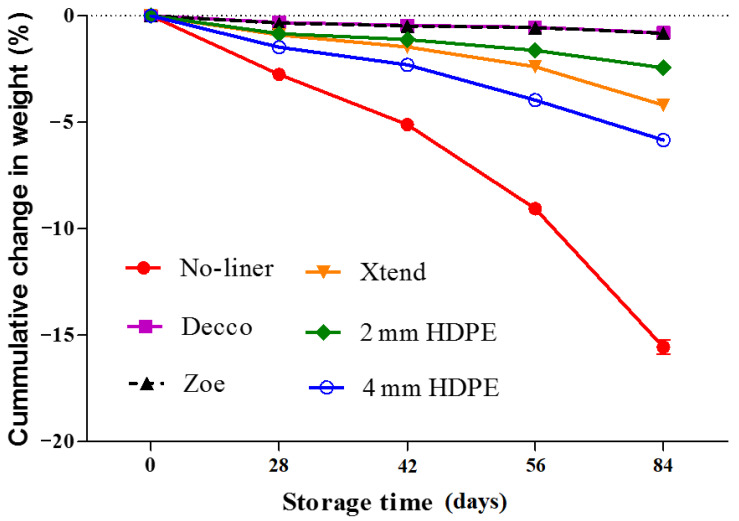
Cumulative change in weight during prolonged cold storage of pomegranate fruit (cv. Wonderful) at 5 °C and 90% relative humidity (RH). HDPE: high density polyethylene.

Moisture loss is the major contributor to weight loss of harvested fruit during postharvest handling. Other physiological activities such as respiration can contribute to mass loss through the utilisation of fruit contents such as the carbohydrates in generating energy to support life processes of the fruit [38,39,40]. During storage, fruit packed with no-liner lost more weight than fruit packed in liners. At the end of 84 d of cold storage, the no-liner packed fruit lost 15.6 ± 0.3% of initial weight (Figure 8). However, fruit packed in non-perforated ‘Decco’ and ‘Zoe’ liners lost only 0.79 and 0.82%, respectively. Fruit packed in micro-perforated Xtend^®^ liners lost 4.17%, compared to 2.44 and 4.17% by fruit packed in 2 mm macro-perforated HDPE and 4 mm macro-perforated HDPE liners, respectively. Non-perforated (‘Decco’ and ‘Zoe’) liners minimised fruit weight loss by 94.0% compared to micro-perforated Xtend^®^ (73.2%), 2 mm macro-perforated HDPE (84.3%), and 4 mm macro-perforated HDPE (62.5%) liners. Weight loss increased with increasing ventilation area of the liners, as observed in kiwifruit [41]. The impact of liners on weight loss can be attributed to the fact that liners act as barriers to the moisture exchange between the immediate environment of the fruit inside liners and the outside environment. A high RH around the fruit minimises moisture loss from the fruit [42]. Liners maintain a high RH around the fruit, reducing the difference in vapor pressure inside the skin surface and immediate surrounding, hence reducing moisture diffusion [36]. Similar to our results, packing pomegranate (cv. Hicrannar) in MAP liners minimised fruit weight loss to 1.5 and 4.4% compared to 17.2% for fruit packed with no-liner, after 120 d of storage at 6 °C [22]. Al-Mughrabi et al. [43] observed 18.3% average weight loss for pomegranate fruit (‘Taeifi’, ‘Banati’, and ‘Manfaloti’ cultivars) packed in plastic crates only (without liners) for 42 d at 5 °C. Storing pomegranate (cv. Wonderful) fruit in MAP liners and shrink wraps maintained a weight loss less than 2% throughout storage period of 4 months, compared to 16.5% for fruit packed with no-liner after 90 days of storage at 7 °C [20]. Mukama et al. [44] reported that pomegranates packed in ventilated cartons without liners had a 17.5% more moisture loss than fruit packed in liners. Critical limits of weight loss in fresh fruit are scarce [45]. A weight loss of 5% can initiate shrivelling in pomegranates [38], which negatively affects fruit marketability. However, weight loss in pomegranates is majorly from the peel portion of the fruit [46] and therefore the arils (edible portion) remains largely preserved for consumption and use in juice processing.

#### 3.2.2. Fruit Lineal Size

The loss in moisture and weight leads to loss in fruit length, diameter, fruit circumference, and sphericity, which may lead to shrivelling, shrinkage, and loss in visual appeal. Generally, all liner treatments minimised loss in fruit length, diameter, and circumference compared to the no-liner treatment throughout the storage period. The non-perforated ‘Decco’ and ‘Zoe’ liners were significantly better in minimising loss in fruit length, diameter, and circumference compared to micro-perforated Xtend^®^ and 2 and 4 mm macro-perforated HDPE liners (Table 3).

At the end of the 84 d of cold storage, fruit in no-liner lost 8.6% of the initial fruit length, while fruit packed in non-perforated ‘Decco’ and ‘Zoe’ liners lost 1.2% and 1.0% in fruit length, respectively. Fruit in micro-perforated Xtend^®^ liners lost 2.7% of initial fruit length compared with 3.4 and 5.1% for fruit packed in 2 and 4 mm macro-perforated HDPE liners, respectively. A similar pattern was observed for loss in fruit diameter, where fruit packed with no-liner lost 5.4% compared to 1.1 and 0.8% for fruit packed with non-perforated ‘Decco’ and ‘Zoe’ liners, respectively. Micro-perforated Xtend^®^, 2 mm macro-perforated HDPE, and 4 mm macro-perforated HDPE liners minimised loss in fruit diameter to 2.2, 2.1, and 3.7%, respectively. A reduction in fruit circumference is a direct indicator of fruit shrinkage. After 84 d of cold storage, fruit packed with no-liner lost 4.1% of their initial circumference, compared to 1.0% and 0.8% for fruit packed in non-perforated ‘Decco’ and ‘Zoe’ liners, respectively. Perforated liners minimised the loss in fruit circumference to about half the loss in no-liner. Fruit packed with micro-perforated Xtend^®^ liners lost 2.3% of their initial circumference compared to 1.8 and 2.8% for fruit packed in 2 and 4 mm macro-perforated HDPE liners, respectively.

Generally, the loss was more in fruit length than in fruit diameter. Shrivelling was more concentrated on the base of the fruit than on the sides. Quite similar results observed by Al-Mughrabi et al. [43] on different pomegranate cultivars conventionally stored in plastic boxes at storage temperatures of 5 °C, 10 °C, and ambient temperature for 56 d. The authors observed that the loss in fruit length and diameter is influenced by storage time, temperature, and cultivar. In their study, the cv. ‘Manfaloti’ with relatively lower fruit weight loss also registered lower loss in fruit diameter and length, as compared to cv. ‘Banati’.

#### 3.2.3. Peel Thickness

**Figure 9:**
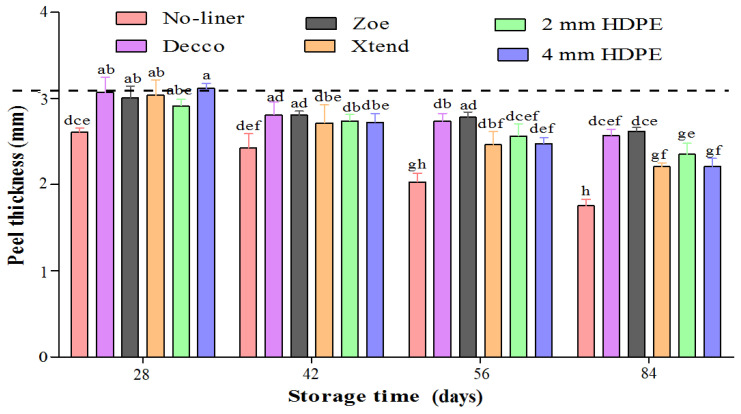
Peel thickness of pomegranate (cv. Wonderful) fruit stored at 5 °C and 90% relative humidity (RH). Mean values (vertical bars) with different letters are significantly different (*p* < 0.05) according to Duncan’s multiple range test. HDPE: high density polyethylene.

The dynamics of moisture loss of fruit may influence each of the fruit fractions differently. The porous nature and position of the pomegranate fruit skin makes it so prone to moisture loss because it comes into direct contact with the surrounding. Moisture loss in pomegranate fruit is primarily from the peel resulting in a reduction in peel thickness [5,44]. The greatest loss in peel thickness was observed in fruit packed with no-liner. Fruit packed in non-perforated liners retained more peel thickness than fruit packed in perforated liners (Figure 9). After 84 d of cold storage, fruit packed with no-liner lost 41.8% of the initial peel thickness. However, non-perforated ‘Decco’ and ‘Zoe’ liners minimised the loss in fruit peel thickness to 14.8 and 13.2%, respectively. Fruit lost 26.8% peel thickness when packed in micro-perforated Xtend^®^ liners, and 22.0 and 26.7% in 2 mm macro-perforated and 4 mm macro-perforated HDPE liners, respectively. Similarly, Arendse et al. [5] reported a decrease in peel thickness with storage time of pomegranate (cv. Wonderful) packed in conventional corrugated boxes and stored at different temperatures (5, 7.5, 10, and 21 °C). The authors attributed the drastic decrease in peel thickness to low RH and high temperature (21 °C). The thicker peel of fruit packed with non-perforated liners can be attributed to higher RH inside bags compared to fruit packed with perforated liners.

#### 3.2.4. Fruit Shrivelling

**Figure 10:**
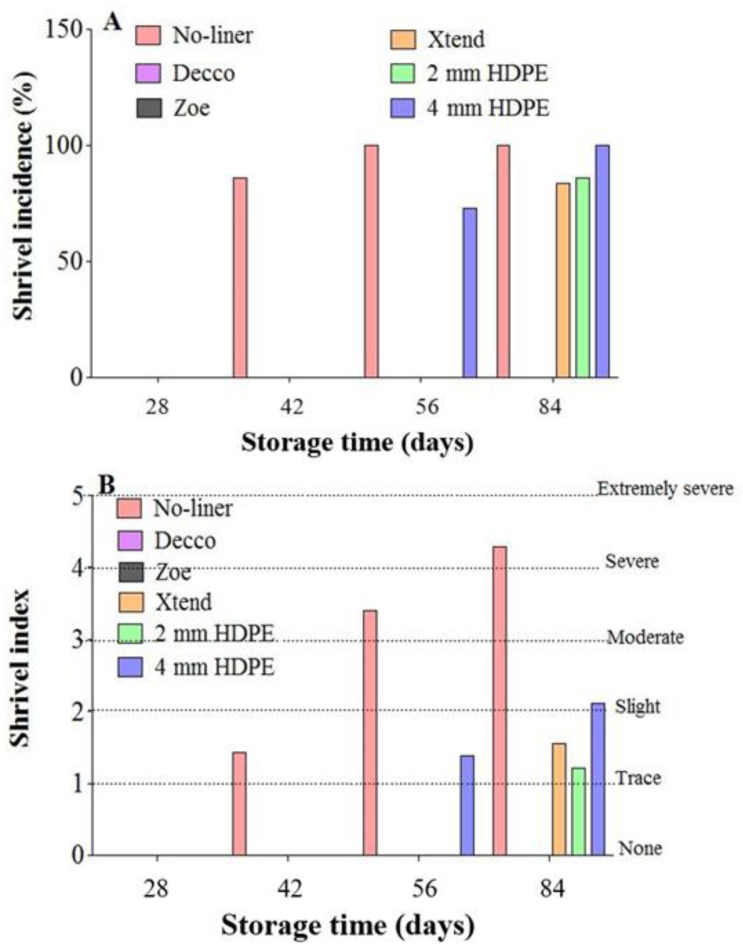
Percentage of total shrivelled fruit (shrivel incidence) (**A**) and shrivel index (incidence) (**B**) observed on pomegranate fruit stored for 84 d at 5 °C and 90% relative humidity (RH). HDPE: high density polyethylene.

The effect of liner packaging on fruit shrivelling is summarised in Figure 10A,B. Fruit shrivelling results from moisture loss and subsequent loss in cell turgor pressure [47]. In pomegranates, shrivelling is expected after a 5% loss in fruit weight [38]. Fruit shrivelling was evident at 42 d of cold storage after 5.1% loss in weight for fruit packed with no-liner, with 86.1% incidences of shrivelling (Figure 10A). At 56 d of cold storage, shrivel incidence increased to 100% for fruit packed with no-liner. However, there was no incidences of fruit shrivelling observed for fruit packed with non-perforated ‘Decco’ and ‘Zoe’ liners throughout 84 d of storage. Slight shrivelling was observed especially at the crown area for fruit packed with micro-perforated Xtend^®^ and 2 mm macro-perforated HDPE liners after 84 d of storage, with a shrivel incidence of 83.3 and 85.7%, respectively. However, shrivelling started at 56 d for fruit packed with 4 mm macro-perforated HDPE liners with an incidence of 72.7%.

The severity of fruit shrivelling (shrivel index) increased with storage time (Figure 10B). At 84 d of storage, fruit packed with no-liner were severely shrivelled with a shrivelling index of 4.3 (86.0%) compared to cases of extreme shrivelling with an index of 5 (100%). However, fruit packed with micro-perforated Xtend^®^ and 2 mm macro-perforated HDPE liners were tracely shrivelled with shrivel index of 1.6 (31.1%) and 1.2 (24.3%), respectively. Fruit packed with 4 mm macro-perforated HDPE liners were slightly shrivelled, having a shrivel index of 2.1 (42.0%). The high shrivel incidence and index in fruit packed with no-liner is attributed to excessive moisture loss during storage. Plastic liners, due to their barrier ability, maintain high relative humidity around the fruit, minimising moisture loss and subsequent shrivelling. Wiley et al. [41] did not observe shrivelling in kiwifruit packed in non-perforated and macro-perforated liners, but reported shrivelling for fruit packed with micro-perforated liners, after 119 d storage at 0 °C.

### 3.3. Respiration Rate

**Figure 11:**
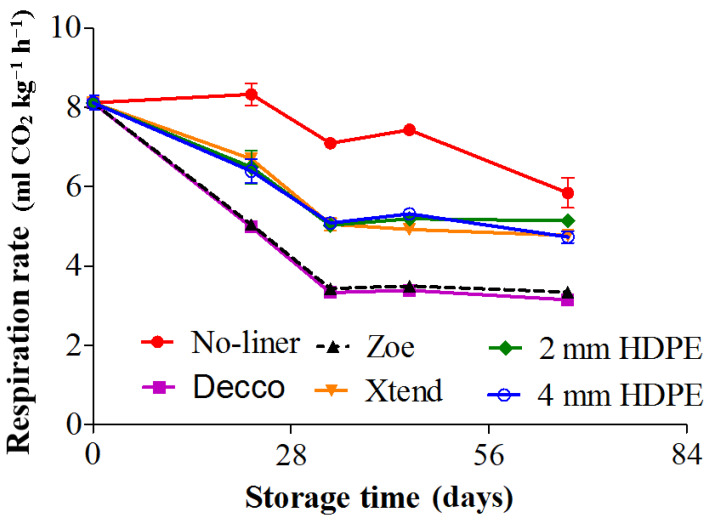
Respiration rate of pomegranate fruit determined by a closed system at 5 °C and 90% relative humidity (RH). HDPE: high density polyethylene.

Respiration rate (RR) of pomegranates (non-climacteric fruit) was generally low, and the decrease with storage period (Figure 11) may be attributed to senescence after harvest. Throughout the storage period, respiration rate was highest in fruit packed with no-liner, followed by fruit packed with perforated liners and lowest in fruit packed with non-perforated liners. Respiration rate for fruit packed in non-perforated liners decreased from 8.1 to about 3.3 mL CO_2_ kg^−1^ h^−1^ within 42 d of cold storage and remained stable to the end of storage. Mphahlele et al. [20] reports quite similar trend for pomegranate (cv. Wonderful) packed in MAP liners, where RR decreased within 28 d and stayed stable throughout 84 d of storage at 7 °C. The authors observed higher RR in control fruit than fruit packed with MAP at the end of 3 months. The initial respiration rate of fruit before storage decreased by 28.4% at the end of 84 d of storage for fruit packed with no-liner, compared to 61.7 and 59.3% for fruit packed in non-perforated ‘Decco’ and ‘Zoe’ liners, respectively. Micro-perforated Xtend^®^ and 4 mm macro-perforated HDPE liners reduced respiration rate of the fruit by 42.0% compared to 37.0% by 2 mm macro-perforated HDPE liners.

Other researchers also reported a decline in respiration rate with storage time for pomegranate fruit [6,48]. Passive MAP achieved by non-perforated ‘Decco’ and ‘Zoe’ liners is probably responsible for the low respiration rate. Nanda et al. [8] reported that MAP inform of shrink-wrapping reduced respiration rate of pomegranate, attributing it to the ability of the films having a low permeability to gases. Furthermore, the lower RR in fruit packed with non-perforated and perforated liners compared to fruit packed with no-liner can be attributed to alleviation of water stress from around the fruit [49].

### 3.4. Textural Properties

#### 3.4.1. Fruit Puncture Resistance

The ability of harvested fruit to resist a puncturing force provides information on the structural integrity. There was a decline in fruit puncture resistance with storage time for all treatments. The baseline (initial) fruit texture was best retained by fruit packed with non-perforated liners followed by fruit packed in perforated liners and no-liner packaging. At the end of 84 d of cold storage, fruit packed with no-liner lost 28.3% of the initial whole fruit firmness (116.1 ± 2.0 N). However, packing fruit in non-perforated ‘Decco’ and ‘Zoe’ liners reduced fruit firmness by 8.0% and 6.8%, respectively. Micro-perforated Xtend^®^ liners minimised fruit firmness by 12.0% compared to 15.8% and 15.5% by 2 mm macro-perforated HDPE and 4 mm macro-perforated HDPE liners, respectively (Table 4). The general decline in texture with storage time can be attributed to fruit softening resulting from enzymatic disintegration of cell wall structure [50]. Similar results reported by Mansouri et al. [51], and Arendese et al. [5] reported declines in whole fruit firmness with storage time for different conventionally packed pomegranate fruit (cv. Hondos-e-Yalabad, Malas-e-Saveh, and Wonderful) in boxes. The higher respiration rate observed in fruit packed with no-liner and macro-perforated liners may have contributed to the higher loss in fruit texture, compared to fruit packed in passively modified atmosphere by non-perforated liners. Drake et al. [52] observed that ‘Bartlett’ pears at low temperatures of 1 °C packed in MAP liners retained more fruit firmness than pear packed under regular atmosphere. The authors reported that storing pears under controlled atmosphere retained fruit firmness throughout cold storage, irrespective of packaging treatment. Similar to our results, Kumar et al. [37] reported that pomegranate (cv. ‘Baghwa’) packed in Xtend^®^ MAP liners retained better and desirable firmness compared to fruit packed with polypropylene liners and with no-liner stored at 4 °C for 120 d.

#### 3.4.2. Aril Firmness

Generally, aril firmness increased in fruit packed with no-liner compared to decreasing aril firmness in fruit packed with liners (Table 4). The increase in aril firmness for fruit packed with no-liner could be attributed to moisture loss, leading to hardening of aril tissues. The decrease in aril firmness is often associated to quality deterioration and may be due to physiological activity such as respiration that brings about softening and disintegration of cell wall structure [50,53]. There was no significant difference in aril firmness for fruit packed with liners throughout the storage period. Fruit packed with non-perforated ‘Decco’ and ‘Zoe’ liners retained more aril firmness compared to fruit packed with perforated liners. At the end of 84 d of storage, fruit packed in either of the non-perforated (‘Decco’ and ‘Zoe’) liners lost 2.0% of initial aril firmness (143.9 ± 1.5 N), compared to 2.8, 5.5, and 3.5% for fruit in micro-perforated Xtend^®^, 2 mm macro-perforated HDPE liners, and 4 mm macro-perforated HDPE liners, respectively. Liners have been reported to maintain desirable firmness in pomegranate and table grape [36,37]. Similar results have been reported with the application of heat shrinkable films on pomegranate fruit [8,17].

### 3.5. Fruit Decay

**Figure 12:**
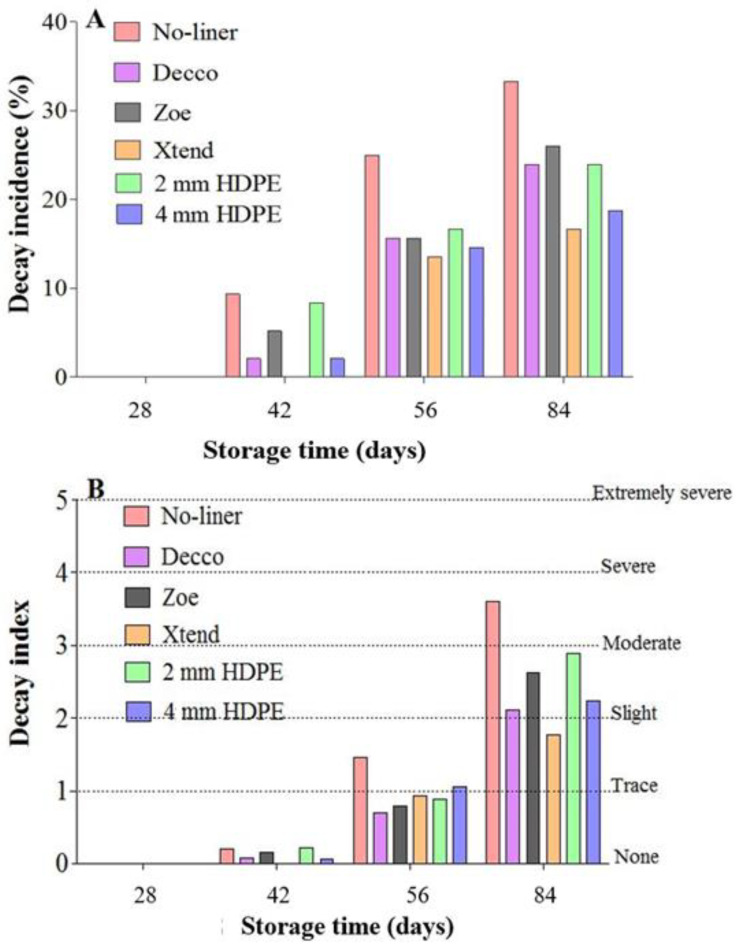
(**A**) Percentage cumulative decay incidence. (**B**) Cumulative decay index (severity). Pomegranate fruit stored for 84 d at 5 °C and 90% relative humidity (RH). HDPE: high density polyethylene.

The incidence of decayed fruit increased with storage time in all treatments. A similar trend was observed in pomegranate cultivars ‘Mollar de Elche’ and ‘Wonderful’ stored at 6 °C and 7 °C, respectively [20,54]. At the end of 84 d of cold storage, 35.4% of fruit packed with no-liner were lost to visible mould. However, packing fruit in non-perforated ‘Decco’ and ‘Zoe’ liners minimised decay incidence to 24.0 and 26.0%, respectively. Furthermore, packing fruit in micro-perforated Xtend^®^ liners minimised fruit decay incidence to 17.7%, compared to 24.0 and 18.5% for fruit packed in 2 mm macro-perforated and 4 mm macro-perforated HDPE liners, respectively (Figure 12A). Selcuk and Erkan [22] reported similar results on ‘Hicrannar’ pomegranate stored at 6 °C for 120 d, where the no-liner control registered 40% decay compared to 13.3 and 26.7% for MAP liner treatments. On the contrary, Laribi et al. [54] and Mphahlele et al. [20] reported higher decay incidence in pomegranate (cv. ‘Mollar de Elche’ and ‘Wonderful’) packed with MAP liners than with no-liners at the end of 84 and 140 d of cold storage, respectively. However, no significant difference in decay incidence between shrink-wrapped and non-wrapped pomegranate (cv. ‘Primosole) at 70 d of cold storage was reported by D’Aquino et al. [17]. The higher decay incidence of fruit packed in non-perforated liners could be attributed to higher moisture condensation within liner bags and lower WVTR across film, resulting into accelerated fruit moulding compared to fruit packed in perforated liners (Figure 12A).

Fruit decay severity provides insight into the extent of the decay on a particular fruit. The influence of packaging treatments on fruit decay severity was different from their influence on decay incidence. Fruit packed with no-liner had the highest decay severity index than fruit packed in liners. The severity (index) of decay was higher in fruit packed with perforated liners compared to fruit packed in non-perforated liners (Figure 12B). This could be attributed to a lower respiration rate observed in fruit packed with non-perforated ‘Decco’ and ‘Zoe’ liners compared to fruit packed in perforated liners. Selcuk and Erkan [22] reported a no significant difference in decay index for control treatment and MAP liner treatments for pomegranate stored at 6 °C for 120 d.

### 3.6. Colour Attributes

#### 3.6.1. Fruit Peel Colour

Fruit peel colour is an important contributor to visual appeal and acceptance of pomegranate fruit by consumers. Generally, there was a progressive decline in the lightness (L*) values with storage time (Table 5). Fruit skin lightness was 51.7 ± 2.4 before storage, after 84 d of storage; fruit packed with no-liner lost 30.0% of the lightness. This can be attributed to excessive moisture loss causing the peel to become pale. The darkening (L*→ 0) of the peel is expected as the fruit ages during storage. However, packing fruit with non-perforated ‘Decco’ and ‘Zoe’ liners significantly (*p* < 0.05) minimised the loss in skin colour lightness to 5.7% and 3.6%, respectively. Fruit packed with micro-perforated Xtend^®^ lost 14.1% compared to 15.0% and 15.7% for fruit packed with 2 mm macro-perforated and 4 mm macro-perforated HDPE liners, respectively. The difference in results can be attributed to differences in the ability of liners to minimise moisture loss and respiration rate. Similarly, Selcuk and Erkan [22] reported higher skin colour lightness for pomegranate (cv. Hicrannar) fruit stored under MAP liners with the fruit looking brighter and fresher compared to the no liner control fruit at the end of 4 months of cold storage and additional 3 d of shelf life.

There was no difference in peel redness colour (a*) among treatments throughout the study; however at 84 d, fruit packed with micro-perforated Xtend^®^ and non-perforated ‘Decco’ and ‘Zoe’ liners retained the initial skin redness colour (a*) before storage. However, Drake [52] reported that packing pears in MAP liners preserved more of the green colour at 90 d of cold storage than did the pears under regular atmosphere at 30 d of storage. This could be attributed to the ability of liners modifying the atmosphere around the fruit, thereby minimising break down of colour pigments.

The effect of storage time on chroma (C*) was only significant on fruit packed with no-liner and macro-perforated HDPE liners. At 84 d of cold storage, fruit packed with non-perforated ‘Decco’, non-perforated ‘Zoe’, and micro-perforated Xtend^®^ liners significantly retained the initial skin C* (colour saturation) compared with fruit in other treatments. Furthermore, fruit packed with macro-perforated HDPE liners significantly retained higher C* than fruit packed with no-liner. Selcuk and Erkan [22] reported a no significant impact of liner packaging on the chroma (C*) for ‘Hicrannar’ pomegranate stored for 120 d at 6 °C. A decrease in skin colour parameters L* and C* was observed with minimal changes for wrapped fruit compared to un-wrapped pomegranate (cv. Primosole) stored at 8 °C for 84 d storage [17].

#### 3.6.2. Aril Colour

The colour of arils is very important, especially in terms of the consumption of fresh pomegranate fruit. There was a significant effect of storage time on lightness (L*), redness (a*), and chroma (C*) colour attributes of arils for all treatments (Table 6). Fruit packed with liners significantly retained higher L* and a* aril colour attributes in comparison with fruit packed with no-liner at 84 d of cold storage. Fruit packed with no-liner retained 55.7% of the initial aril L* colour attribute, compared to 82.4 and 76.9% for fruit packed with non-perforated ‘Decco’ and ‘Zoe’ liners, respectively. Fruit packed in micro-perforated Xtend^®^ liner retained 67.3% of aril L* colour attribute, with no significant difference compared to 68.4% and 70.4% for fruit packed with 2 mm macro-perforated and 4 mm macro-perforated HDPE liners, respectively. These results could be attributed to the influence of liner packaging on fruit weight loss and respiration rate. Excessive loss of moisture and high respiration rate by the no-liner packed fruit could have resulted in the loss of aril colour lightness and redness due to water stress and degradation of colour pigments.

There was no significant difference in a* and C* aril colour attributes among all fruit packed with liners (Table 6). Therefore, perforation of liners did not have an effect on the redness and tone saturation colour attributes of the arils. Similarly, Arendse et al. [5] observed significant decrease of aril colour parameters L*, a*, and C* with storage time for pomegranate (cv. ‘Wonderful’) fruit packed in boxes and stored at different temperature conditions.

### 3.7. Principal Component Analysis

**Figure 13:**
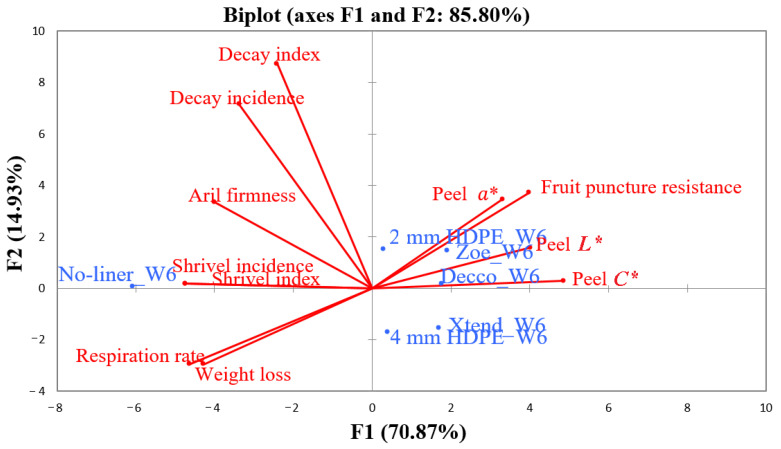
Principal component analysis of the first two factors (F1 and F2) due to physical and physiological attributes of pomegranate (cv. Wonderful) after 42 d of storage at 5 °C and 95% relative humidity (RH). W6 = 42 d. L*: lightness index describes surface color in the range from 0 (black) to 100 (white); a*: redness index describes surface color in the range from green (−a*) to red (+a*); C*: chroma; HDPE: high density polyethylene.

**Figure 14:**
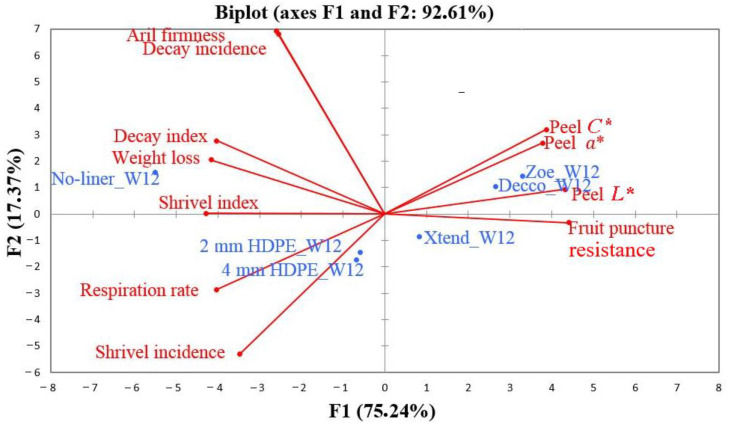
Principal component analysis of the first two factors (F1 and F2) due to physical and physiological attributes of pomegranate (cv. Wonderful) after 84 d of storage at 5 °C and 95% relative humidity (RH). W12 = 84 d. L*: lightness index describes surface color in the range from 0 (black) to 100 (white); a*: redness index describes surface color in the range from green (−a*) to red (+a*); C*: chroma; HDPE: high density polyethylene.

The averages of quality attributes of pomegranate fruit packed with no-liner, non-perforated ‘Decco’ and ‘Zoe’, micro-perforated Xtend^®^, and macro-perforated 2 and 4 mm HDPE liners are shown in Figure 13 and Figure 14. Total variability was explained by five principal factors. Shipping fruit takes 42 d across the Atlantic Ocean from South Africa to Europe, which is the main export market. After 42 d of storage, the first two principal factors (F1 and F2) explained 85.8% of the total variability. Along F1 (explaining 70.9% of total variability), packaging fruit with no-liner was associated with higher weight loss, shrivelling, high respiration rate, and aril hardening by 42 d of storage. The same attributes associated with no-liner packaging had high negative values along F1 (Table 7). On the other hand, packing fruit with both non-perforated and perforated liners was associated with retaining fruit puncture resistance and peel colour attributes of L*, C*, and a*. The same attributes associated with liner packaging had high positive values along F1 (Table 7). Along F2 (explaining 14.9% of total variability), packing fruit with no-liner, non-perforated ‘Decco’ and ‘Zoe’, and macro-perforated 2 mm HDPE were associated to facilitating fruit decay (incidence and index). Variables of decay incidence and index had high positive values along F2 (Table 7).

After 84 d of storage, a clearer separation between packaging treatments was observed (Figure 14). In this case, the first two component factors (F1 and F2) explained 92.6% of the total variability, with F1 and F2 accounting for 75.2 and 17.4%, respectively. Along F1, packaging fruit with no-liner and macro-perforated 2 and 4 mm HDPE liners was associated with facilitating fruit weight loss, shrivelling (incidence and index), respiration rate, and decay index. On the other hand, packing fruit with non-perforated ‘Decco’ and ‘Zoe’ and micro-perforated Xtend^®^ liners was associated with retaining fruit puncture resistance and peel colour attributes of L*, C*, and a*. Along F2, packing fruit with no-liner and non-perforated ‘Decco’ and ‘Zoe’ liners was associated with decay incidence and aril firmness (or hardness as applicable to no-liner packed fruit).

## 4. Conclusions

High incidence of postharvest fruit losses and waste is a major problem facing the pomegranate industry worldwide [55,56]. The use of plastic liners as internal packages in the multi-scale packaging of pomegranate fruit plays a major role in minimising quantitative and qualitative losses during prolonged cold storage. Packaging pomegranate (cv. Wonderful) in non-perforated liners greatly minimises mass loss and maintains fruit colour and textural quality during cold storage for 84 d at 5 °C. Micro-perforated Xtend^®^ and 4 mm macro-perforated HDPE liners were able to minimise moisture condensation within the bags and reduced decay incidence, which are some of the challenges of packing fruit in non-perforated liners. Packing fruit with perforated liners also greatly minimised fruit mass and size loss and retained acceptable quality during prolonged storage compared to packing fruit with no-liner. Using micro-perforated Xtend^®^ and macro-perforated 4 mm HDPE can be considered to minimise postharvest losses often associated with inadequate environment control inside packaging compared to the use of non-perforated liners (note that 4 mm HDPE liners are over three times cheaper than micro-perforated Xtend^®^ liners).

## Figures and Tables

**Table 1:** Rate of moisture condensation and corresponding weight loss for 12 pomegranate fruit inside plastic liner bags, at 5 °C and 90% relative humidity (RH). HDPE: high density polyethylene.

Treatment	Weight Loss (g d^−1^)	Condensation Rate (g d^−1^)
Xtend	5.55	2.32
Decco	4.27	3.37
Zoe	3.46	2.85
2 mm HDPE	5.50	3.49
4 mm HDPE	6.81	2.48

**Table 2:** Condensate characterisation inside plastic liner bags for pomegranate fruit stored at 5 °C and 90% relative humidity (RH). HDPE: high density polyethylene.

Treatment	Condensation Score (0–10) ^1^	Condensate Characteristics
Xtend	3.47	Large droplets.
		Condensate entirely on the inside-top wall of the liner.
		Droplets uniformly distributed on top wall.
		Very little condensate in the bottom corner.
		No condensation on fruit.
Decco	5.67	Medium droplets.
		Condensate on both the top and side walls within the liner.
		Droplets uniformly distributed on the walls.
		Visible condensate droplets on the fruit.
Zoe	5.33	Medium to large droplets.
		Condensate on both the top and side walls within the liner.
		Droplets non-uniformly distributed, creating a patch-like pattern
		Visible condensate droplets on the fruit.
2 mm HDPE	4.03	Very tinny/misty droplets on top of the bag.
		No condensation on the fruit and immediate area around perforations.
		Uniformly distributed.
4 mm HDPE	3.50	Very tinny/misty droplets on top of the bag.
		No condensation on the fruit and immediate area around perforations.
		Uniformly distributed.

^1^ Condensation was scored using 0–10 score scale (where 0 = none; 1–2 = trace; 3–4 = slight; 5–6 = moderate; 7–8 = severe; 9–10 = extremely severe).

**Table 3:** Effect of plastic liner treatment on cumulative loss in fruit length, diameter, and circumference of pomegranate (cv. Wonderful) fruit stored at 5 °C and 90% relative humidity (RH).

		Cumulative Loss (%)		
Time (d)	Treatment	Length	Diameter	Circumference
28	No-liner	2.13 ± 0.07 ^def^	1.67 ± 0.17 ^def^	1.07 ± 0.18 ^jg^
	Decco	0.64 ± 0.13 ^ih^	0.34 ± 0.06 ^jk^	0.38 ± 0.09 ^l^
	Zoe	0.45 ± 0.07 ^i^	0.23 ± 0.06 ^k^	0.36 ± 0.07 ^l^
	Xtend	1.40 ± 0.37 ^geh^	0.76 ± 0.17 ^jkh^	0.81 ± 0.07 ^jl^
	2 mm HDPE	0.69 ± 0.18 ^gi^	0.84 ± 0.07 ^jh^	0.55 ± 0.09 ^lk^
	4 mm HDPE	1.52 ± 0.21 ^ge^	1.67 ± 0.16 ^def^	0.86 ± 0.12 ^jli^
42	No-liner	3.85 ± 0.13 ^c^	3.01 ± 0.12 ^c^	2.10 ± 0.08 ^cd^
	Decco	1.11 ± 0.30 ^gi4^	0.65 ± 0.18 ^jki^	0.71 ± 0.11 ^jl^
	Zoe	0.62 ± 0.07 ^ih^	0.39 ± 0.09 ^jk^	0.68 ± 0.14 ^jl^
	Xtend	2.20 ± 0.64 ^de^	1.19 ± 0.22 ^gfhi^	1.42 ± 0.16 ^fegh^
	2 mm HDPE	1.50 ± 0.08 ^ge^	1.30 ± 0.10 ^gfh^	0.95 ± 0.19 ^jhk^
	4 mm HDPE	2.79 ± 0.12 ^d^	2.04 ± 0.11 ^de^	1.50 ± 0.12 ^feg^
56	No-liner	5.39 ± 0.12 ^b^	3.74 ± 0.18 ^b^	2.93 ± 0.16 ^b^
	Decco	1.34 ± 0.34 ^gfh^	0.84 ± 0.15 ^jh^	0.86 ± 0.11 ^jli^
	Zoe	0.78 ± 0.06 ^gi^	0.50 ± 0.17 ^jk^	0.84 ± 0.16 ^jli^
	Xtend	2.81 ± 0.15 ^d^	1.58 ± 0.25 ^dg^	1.88 ± 0.14 ^cde^
	2 mm HDPE	2.06 ± 0.06 ^def^	1.50 ± 0.12 ^ge^	1.34 ± 0.24 ^fghi^
	4 mm HDPE	3.56 ± 0.14 ^c^	2.74 ± 0.10 ^c^	2.07 ± 0.15 ^cd^
84	No-liner	7.41 ± 0.14 ^a^	5.27 ± 0.08 ^a^	4.10 ± 0.17 ^a^
	Decco	1.34 ± 0.32 ^gfh^	1.09 ± 0.17 ^ghi^	1.01 ± 0.10 ^jhk^
	Zoe	0.98 ± 0.08 ^gi^	0.82 ± 0.18 ^jh^	0.89 ± 0.01 ^jik^
	Xtend	3.87 ± 0.49 ^c^	2.64 ± 0.47 ^c^	2.34 ± 0.04 ^c^
	2 mm HDPE	2.67 ± 0.10 ^d^	2.09 ± 0.11 ^d^	1.73 ± 0.13 ^fd^
	4 mm HDPE	4.93 ± 0.24 ^b^	3.71 ± 0.10 ^b^	2.83 ± 0.03 ^b^

Results presented as mean ± S.E. Different letter(s) on column per liner treatment indicate statistically significant differences (*p* < 0.05) according to Duncan’s multiple range test. HDPE: high density polyethylene.

**Table 4:** Whole fruit puncture resistance and aril firmness for pomegranate fruit packed in different liner bags for 84 d at 5 °C and 90% relative humidity (RH).

Storage Time (d)	Treatment	Whole Fruit Puncture Resistance (N)	Aril Firmness (N)
0		116.11 ± 1.96 ^ab^	143.91 ± 1.51 ^dce^
28	No-liner	119.66 ± 2.99 ^a^	146.43 ± 1.96 ^db^
	Decco	114.54 ± 2.19 ^abc^	143.11 ± 2.20 ^de^
	Zoe	115.008 ± 1.90 ^abc^	143.39 ± 3.93 ^de^
	Xtend	114.51 ± 2.46 ^abcd^	142.22 ± 2.11 ^df^
	2 mm HDPE	113.79 ± 2.62 ^abcd^	141.87 ± 1.69 ^df^
	4 mm HDPE	114.36 ± 2.30 ^abcd^	140.87 ± 2.01 ^df^
42	No-liner	104.50 ± 2.06 ^hf^	150.67 ± 2.02 ^ab^
	Decco	114.88 ± 1.39 ^abc^	142.84 ± 2.04 ^df^
	Zoe	115.00 ± 1.71 ^abc^	142.98 ± 1.59 ^df^
	Xtend	111.74 ± 1.34 ^eb^	138.26 ± 1.98 ^fe^
	2 mm HDPE	109.86 ± 2.52 ^ebf^	140.06 ± 2.11 ^df^
	4 mm HDPE	106.85 ± 1.61 ^eh^	138.86 ± 1.95 ^fe^
56	No-liner	92.68 ± 1.65 ^i^	153.93 ± 2.27 ^a^
	Decco	113.05 ± 1.36 ^eb^	141.30 ± 1.05 ^df^
	Zoe	113.89 ± 2.17 ^abcg^	142.76 ± 1.51 ^df^
	Xtend	109.30 ± 1.05 ^ecf^	139.84 ± 1.90 ^df^
	2 mm HDPE	102.51 ± 1.97 ^hgi^	138.74 ± 1.25 ^fe^
	4 mm HDPE	101.15 ± 2.03 ^hi^	140.29 ± 1.60 ^df^
84	No-liner	83.30 ± 2.60 ^j^	149.82 ± 1.30 ^abc^
	Decco	106.82 ± 1.16 ^eh^	141.69 ± 1.34 ^df^
	Zoe	108.18 ± 1.50 ^edfg^	141.01 ± 1.53 ^df^
	Xtend	101.39 ± 1.80 ^hi^	139.82 ± 1.16 ^df^
	2 mm HDPE	97.74 ± 1.05 ^ji^	135.98 ± 0.75 ^f^
	4 mm HDPE	98.09 ± 1.43 ^ji^	138.88 ± 1.24 ^fe^

Mean values with different letters are significantly different (*p* < 0.05) according to Duncan’s multiple range test. HDPE: high density polyethylene.

**Table 5:** Impact of liners on pomegranate fruit peel colour parameters. Fruit were stored at 5 °C and 90% relative humidity (RH).

Time (d)	Treatment	L* ^1^	a* ^2^	C* ^3^
0		51.70 ± 2.36 ^a^	29.74 ± 0.34 ^a^	40.72 ± 0.51 ^ab^
28	No-liner	51.39 ± 2.10 ^a^	29.15 ± 0.66 ^ab^	40.56 ± 0.73 ^ab^
	Xtend	50.11 ± 2.36 ^ab^	29.73 ± 1.56 ^a^	40.74 ± 0.98 ^ab^
	Decco	51.32 ± 1.67 ^a^	29.85 ± 1.40 ^a^	40.36 ± 0.78 ^ab^
	Zoe	51.21 ± 2.00 ^a^	29.68 ± 1.80 ^a^	40.91 ± 0.94 ^a^
	2 mm HDPE	50.83 ± 2.78 ^a^	29.71 ± 1.76 ^a^	40.16 ± 1.71 ^ab^
	4 mm HDPE	51.40 ± 1.52 ^a^	29.56 ± 1.08 ^ab^	41.16 ± 0.70 ^a^
42	No-liner	46.22 ± 1.08 ^ad^	27.10 ± 0.66 ^ad^	38.05 ± 0.59 ^db^
	Xtend	48.56 ± 1.72 ^abc^	29.21 ± 1.61 ^ab^	39.98 ± 0.95 ^ab^
	Decco	49.15 ± 1.80 ^abc^	28.33 ± 1.68 ^abc^	39.97 ± 1.02 ^ab^
	Zoe	51.07 ± 2.19 ^a^	28.72 ± 0.42 ^ab^	40.05 ± 0.78 ^ab^
	2 mm HDPE	48.99 ± 2.78 ^abc^	28.95 ± 2.01 ^ab^	39.74 ± 1.37 ^ab^
	4 mm HDPE	50.11 ± 1.10 ^ab^	27.17 ± 0.52 ^ad^	39.67 ± 0.50 ^ab^
56	No-liner	41.68 ± 0.54 ^d^	26.75 ± 0.76 ^ad^	36.45 ± 0.56 ^dec^
	Xtend	46.39 ± 0.99 ^ad^	26.75 ± 0.65 ^ad^	39.41 ± 0.44 ^ab^
	Decco	49.13 ± 1.22 ^abc^	26.33 ± 0.48 ^ad^	39.45 ± 0.48 ^ab^
	Zoe	49.95 ± 2.46 ^ab^	27.02 ± 0.36 ^ad^	39.91 ± 0.49 ^ab^
	2 mm HDPE	46.24 ± 1.09 ^ad^	26.81 ± 0.20 ^ad^	38.01 ± 1.20 ^db^
	4 mm HDPE	46.27 ± 0.85 ^ad^	25.83 ± 0.51 ^db^	38.92 ± 0.73 ^abc^
84	No-liner	36.20 ± 0.85 ^e^	24.23 ± 0.48 ^d^	32.90 ± 0.67 ^e^
	Xtend	44.44 ± 0.58 ^db^	26.43 ± 0.59 ^ad^	38.54 ± 0.56 ^ab^
	Decco	48.75 ± 1.19 ^abc^	26.15 ± 0.48 ^ad^	38.80 ± 0.47 ^abc^
	Zoe	49.84 ± 0.70 ^ab^	26.90 ± 0.68 ^ad^	38.06 ± 0.90 ^abc^
	2 mm HDPE	43.95 ± 0.72 ^dc^	24.91 ± 0.42 ^dc^	35.43 ± 0.66 ^d^
	4 mm HDPE	43.58 ± 0.77 ^dc^	24.47 ± 0.47 ^d^	35.99 ± 0.40 ^d^

^1^ Lightnesss index describes surface color in the range from 0 (black) to 100 (white). ^2^ Redness index describes surface color in the range from green (−a*) to red (+a*). ^3^ Chroma. Mean values with different letters are significantly different (*p* < 0.05) according to Duncan’s multiple range test. HDPE: high density polyethylene.

**Table 6:** Impact of liner treatment on pomegranate aril colour parameters. Fruit were stored for 84 d at 5 °C and 90% relative humidity (RH).

Time (d)	Treatment	L* ^1^	a* ^2^	C* ^3^
0		20.86 ± 0.38 ^abc^	19.19 ± 0.41 ^a^	21.09 ± 0.66 ^a^
28	No-liner	20.80 ± 0.54 ^abc^	18.20 ± 1.55 ^abc^	19.78 ± 1.71 ^abcd^
	Xtend	21.90 ± 0.68 ^a^	18.08 ± 0.46 ^abc^	20.30 ± 0.55 ^abc^
	Decco	21.50 ± 0.51 ^ab^	18.83 ± 0.52 ^ab^	20.33 ± 0.59 ^abc^
	Zoe	21.59 ± 1.06 ^ab^	18.60 ± 0.62 ^ab^	20.66 ± 0.91 ^ab^
	2 mm HDPE	21.09 ± 0.42 ^abc^	18.25 ± 0.83 ^abc^	19.75 ± 1.02 ^abcd^
	4 mm HDPE	21.90 ± 1.44 ^a^	18.30 ± 0.52 ^abc^	19.97 ± 0.39 ^abcd^
42	No-liner	16.56 ± 1.06 ^ei^	15.31 ± 1.08 ^fd^	16.45 ± 1.21 ^ge^
	Xtend	19.04 ± 1.25 ^eb^	17.01 ± 1.31 ^abcde^	18.38 ± 1.42 ^abcdef^
	Decco	19.65 ± 0.39 ^abcd^	15.10 ± 0.91 ^fd^	16.32 ± 1.04 ^ge^
	Zoe	19.35 ± 0.88 ^abcd^	16.43 ± 1.01 ^fb^	17.67 ± 1.16 ^gb^
	2 mm HDPE	18.77 ± 1.06 ^ecf^	14.49 ± 0.67 ^feg^	15.74 ± 0.83 ^geh^
	4 mm HDPE	18.15 ± 0.90 ^edfg^	15.86 ± 0.66 ^fc^	17.06 ± 0.82 ^gd^
56	No-liner	14.02 ± 0.69 ^i^	12.26 ± 0.75 ^hg^	13.47 ± 0.89 ^ih^
	Xtend	16.35 ± 0.40 ^if^	15.80 ± 0.66 ^fc^	17.82 ± 0.62 ^gb^
	Decco	17.42 ± 0.42 ^edfg^	17.16 ± 0.44 ^abcd^	18.30 ± 0.57 ^abcdef^
	Zoe	18.05 ± 0.35 ^edfg^	17.37 ± 0.59 ^abcd^	18.58 ± 0.74 ^abcde^
	2 mm HDPE	17.29 ± 1.46 ^edfg^	16.03 ± 0.83 ^fc^	17.48 ± 0.91 ^fgc^
	4 mm HDPE	15.60 ± 0.63 ^ig^	15.48 ± 0.51 ^fd^	16.95 ± 0.57 ^gd^
84	No-liner	11.62 ± 0.68 ^j^	11.34 ± 0.43 ^h^	12.94 ± 0.62 ^i^
	Xtend	14.14 ± 0.76 ^i^	14.04 ± 0.29 ^fg^	15.24 ± 0.31 ^gi^
	Decco	17.19 ± 0.67 ^edfgh^	16.04 ± 1.06 ^fc^	17.22 ± 1.23 ^gd^
	Zoe	16.04 ± 0.56 ^ig^	16.01 ± 0.72 ^fc^	18.03 ± 0.94 ^gb^
	2 mm HDPE	14.26 ± 0.59 ^i^	14.50 ± 0.58 ^feg^	15.39 ± 0.64 ^gif^
	4 mm HDPE	14.69 ± 0.58 ^ih^	14.46 ± 0.58 ^feg^	16.68 ± 0.83 ^ge^

^1^ Lightnesss index, describes surface color in the range from 0 (black) to 100 (white). ^2^ Redness index, describes surface color in the range from green (−a*) to red (+a*). ^3^ Chroma. Mean values with different letters are significantly different (*p* < 0.05) according to Duncan’s multiple range test. HDPE: high density polyethylene.

**Table 7:** Factor loadings and scores for the first two principal factors (F1 and F2) for pomegranate packed in no-liner and with different liners. HDPE: high density polyethylene.

	42 d of Storage		84 d of Storage	
	F1	F2	F1	F2
Factor Loadings				
Weight loss	−0.955	−0.278	−0.945	0.224
Fruit firmness	0.818	0.349	0.999	−0.036
Aril firmness	−0.824	0.317	−0.588	0.755
Decay incidence	−0.695	0.674	−0.579	0.741
Decay index	−0.496	0.825	−0.913	0.302
Shrivel incidence	−0.972	0.017	−0.791	−0.578
Shrivel index	−0.972	0.017	−0.973	0.003
Respiration rate	−0.879	−0.279	−0.913	−0.312
Peel L*	0.826	0.150	0.979	0.102
Peel a*	0.682	0.328	0.858	0.292
Peel C*	0.998	0.029	0.878	0.348
Factor Scores				
No-liner_W6	−6.070	0.050	−5.506	1.587
Decco_W6	1.782	0.166	2.648	1.042
Zoe_W6	1.898	1.486	3.296	1.419
Xtend_W6	1.709	−1.537	0.821	−0.869
2 mm HDPE_W6	0.300	1.536	−0.584	−1.453
4 mm HDPE_W6	0.380	−1.701	−0.676	−1.727

## Data Availability

Data sharing is contained within the article.
